# Examiner experience moderates reliability of human lower extremity muscle ultrasound measurement – a double blinded measurement error study

**DOI:** 10.1186/s13089-025-00424-6

**Published:** 2025-03-26

**Authors:** Konstantin Warneke, Stanislav D. Siegel, Jonas Drabow, Lars H. Lohmann, Daniel Jochum, Sandro R. Freitas, José Afonso, Andreas Konrad

**Affiliations:** 1 Department for Movement Science and Exercise Physiology, Friedrich Schiller University Jena, Jena, Germany; 2 Institute of Human Movement Science, Sport and Health, University of Graz, Graz, Austria; 3 Institute of Psychology, Leuphana University Lüneburg, Lüneburg, Germany; 4 Department of Health Science and Technology, ETH Zürich, Zürich, Switzerland; 5 Neuromuscular Research Lab, Faculty of Human Kinetics, University of Lisbon, Lisbon, Portugal; 6 Centre of Research, Education, Innovation, and Intervention in Sport (CIFI 2 D), Faculty of Sport, University of Porto, Porto, Portugal

**Keywords:** Sonography, Agreement, Intraclass correlation coefficient, Pennation angle, Muscle thickness

## Abstract

Structural muscle properties are critical in health and athletic settings, with magnetic resonance imaging considered the gold standard assessment procedure under static conditions due to its reliability and objectivity. Practical limitations, including cost and accessibility, have led to the increasing use of ultrasound as an alternative for skeletal muscle morphological parameters. However, ultrasound measurements are sensitive to evaluation conditions and assessor experience, which has not been sufficiently explored, yet. Therefore, this study investigated the influence of assessor experience on the reliability of ultrasound measurements. A double-blind design was used, involving an experienced assessor (> 12,000 images for several years) and multiple inexperienced assessors (< 100 images) to collect data from 39 recreationally active participants. Measurements of muscle architecture were conducted in the leg muscles over two consecutive days, generating 1,248 ultrasound images. Relative and absolute reliability were analyzed using intraclass correlation coefficients (ICCs), standard error of measurement, minimal detectable change, mean absolute error (MAE), mean absolute percentage error (MAPE) and Bland-Altman analyses. Relative reliability was good to excellent in all measurement spots and time-points for muscle thickness (ICC = 0.76–0.98) irrespective of assessor experience, except for the inter-day comparison for the gastrocnemius lateralis by the inexperienced assessors, (ICC = 0.58). The pennation angle assessment ranged from insufficient to excellent reliability (ICC = 0.18–0.94) and experience contributed greatly to better results. The random error for the inexperienced assessors was reflected in two- to three-times higher MAEs/MAPEs and limits of agreement in the Bland-Altman analyses, respectively. The findings emphasize the importance of experience and standardization in achieving reliable ultrasound data, particularly for (a) sensitive parameters like the pennation angle and/or (b) inter-day, intra-subject comparisons.

**Supplementary Information:**

The online version contains supplementary material available at 10.1186/s13089-025-00424-6.

## Introduction

Assessing structural muscle properties is of paramount importance in health-related and athletic settings [37]. As the muscle is the largest metabolically active structure in the human body, increasing muscle mass has several positive effects for health [29], and is associated with increased strength and performance [15]. In contrast, muscle size can also decrease, which can be observed in numerous studies addressing sarcopenia in the elderly [3, 32]. Sarcopenia and hypertrophy are slow processes, leading to small effect sizes over common intervention periods of just a few weeks [9, 10]. For instance, the literature highlights mean increases of muscle size due to a resistance training of about 7–31% when performed for 5–12 weeks depending on the muscle and population being examined [1, 27, 41]. Studies in sarcopenia found reductions in muscle thickness or cross-sectional area averaging 1% per year in a population aged > 50 years [16]. Consequently, sensitive measurement equipment and highly standardized, reliable and valid measurement protocols are needed to detect these small changes in muscle morphology [37].

For this purpose, the magnetic resonance imaging (MRI) is the gold standard due to its excellent reliability and validity, and no active interference from the assessors during the procedure [45]. The major drawbacks of MRI testing are (a) limited access to an MRI measurement unit, (b) expensive measurements and (c) can only be done at specialized facilities meaning they are place-bound and (d) time consuming [4, 45]. While these limitations might be neglectable for clinicians and individual diagnostics, MRI measurements are often infeasible when performing cohort study in a scientific context with large sample sizes or measuring morphology under dynamic conditions. Consequently, cheaper and more accessible alternatives are required.

Unsurprisingly, most studies on muscle hypertrophy and atrophy are performed with ultrasound [39, 41]. Ultrasonographic devices constitute a time-saving and flexible solution to monitor muscle thickness, but also architectural parameters such as the pennation angle (PA) or fascicle length [37]. Ultrasound has been implemented in cross-sectional research, but also in intervention studies addressing muscle hypertrophy after resistance training [39] or stretching interventions [31, 47]. Research has shown that ultrasound measurements can be performed reliably [4, 30]. However, the literature is controversial regarding its validity [30, 45], and there are concerns about the objectivity of ultrasound measurements. Especially highly sensitive parameters such as the pennation angle (PA) might be meaningfully affected by even small variance in the evaluation standardization, e.g., the applied pressure, angle and rotation axis, could moderate results and thus reliability [45]. In contrast, several studies address the inter-assessment reliability/objectivity and provide intraclass correlation coefficients (ICC) ranging from 0.72 to 0.99 to showcase that ultrasound was performed under reliable and objective conditions [5, 18, 34].

However, ICCs do not account for systematic errors (e.g., one assessor measuring systematically higher or lower values) or random errors (i.e., random variance in probe pressure or angle standardization) [2, 19, 28]. Absolute indices such as standard error of measurement (SEM) or the minimal detectable change (MDC)/smallest detectable change (SDC)) are based on the ICC, so their validity seems questionable as well [28].

As reliable evaluations of data are a necessary, but no sufficient condition for establishing the validity of a measurement, the objective of this study was to investigate the influence of experience of the assessor on the reliability and measurement error of ultrasound muscle architecture evaluations. In agreement with Warneke et al. [2, 19, 48], we accounted for relative and absolute reliability, as well as random and systematic measurement errors on inter- and intra-day data.

## Methods

**Fig. 1:**
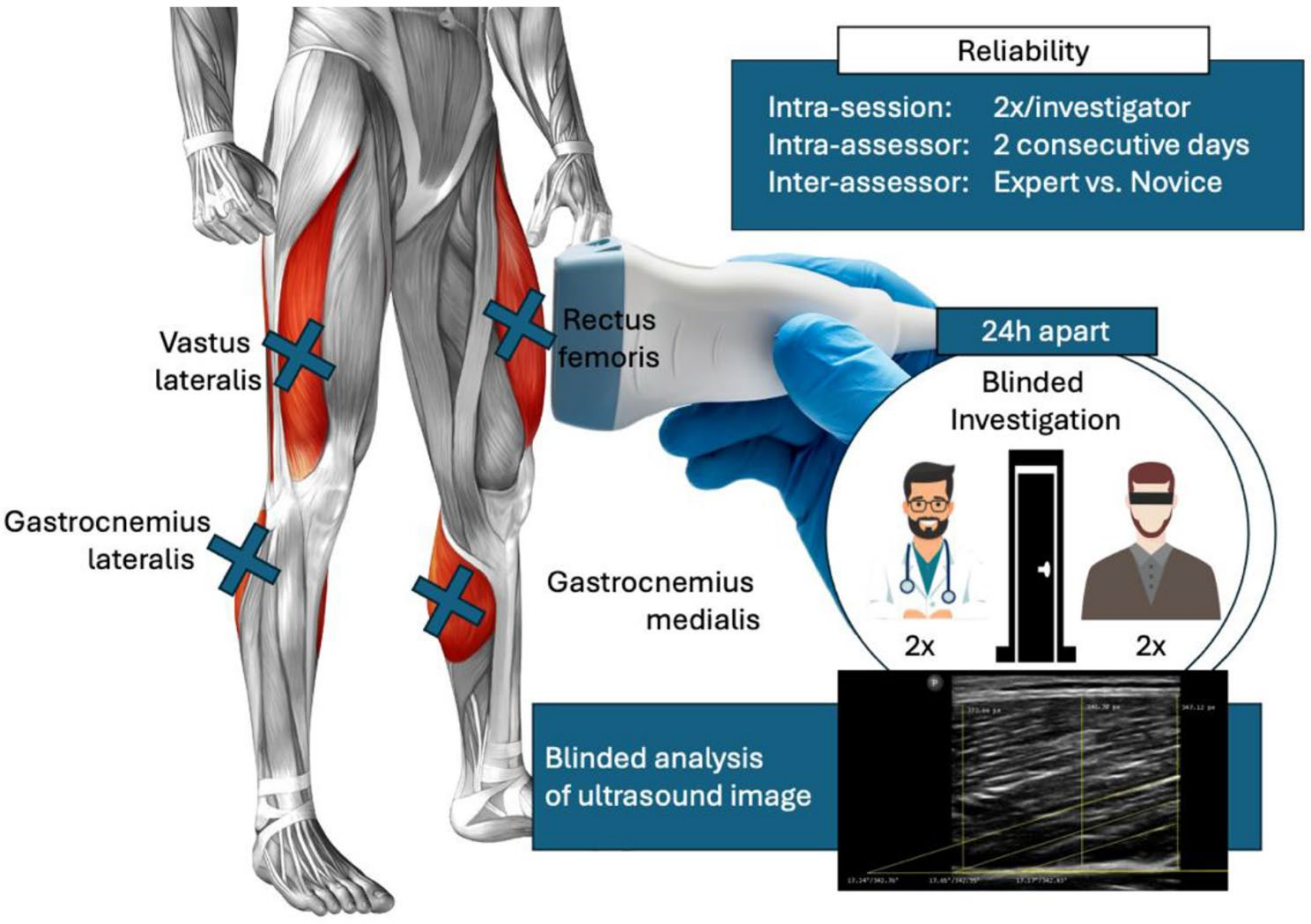
Graphical illustration of the study protocol including image acquisition of an experienced and inexperienced investigator, blinded for the results of the respective other in the quadriceps (rectus femoris and vastus lateralis) and the plantar flexors (lateral and medial head of the gastrocnemius)

The study was designed as a double-blinded (assessors blinded for each others’ results, image assessor blinded for both assessors and participants) reliability study on ultrasound assessments of muscle thickness and PA in the quadriceps and plantar flexors (see Fig. 1). Since previous studies provided concerns dependency on subjective influences of ultrasound results, assessor experience was hypothesized to moderate the precision and accuracy, which areas a vital precondition for scientifically sound muscle ultrasound imaging. To address this issue, the intra- and inter-day reliability was determined in experienced and inexperienced assessors by collecting muscle thickness and pennation angle data from four muscles twice per day (intraday) on two consecutive days (inter-day).

### Participants

No a-priori sample size estimation was performed as this is not available for agreement analyses. However, previous studies used sample sizes of 15 to 29 [5, 11, 22, 33]. To ensure sufficient power and account for potential drop-outs, 39 recreationally active and healthy participants (m: *n* = 20, age = 23.75±2.43 years, height = 179.43±8.68 cm, mass = 78.92±9.92 kg, w = 19, age = 23.91±2.57 years, height = 166.27±5.21 cm, mass = 60.28±6.81 kg) were recruited from the university campus and university sports science program. Participants were considered recreationally active if they participated in a structured sport- or training program at least twice per week for a minimum of 60 min for (at least) one year. To receive comparable results and avoid problems with muscle assessments due to large amounts of fatty tissue, participants with a BMI of > 25 were excluded from the study. All participants were instructed about the study protocol and provided written informed consent. The study was conducted in agreement with the Declaration of Helsinki and was ethically approved by the local ethical review board (No GZ. 39/49/63 ex 2024/25).

### Ultrasound investigation

#### Assessor selection

To investigate the influence of experience on the intra- and inter-day reliability one highly experienced investigator (> 12,000 ultrasound images across multiple published articles over years) and several inexperienced assessors (≤ 100 ultrasound explorations) performed the data collection. Within this study alone, more than 1,000 images were acquired, so we had to rotate the inexperienced assessor randomly within our pool of exercise and physical education students. This procedure was performed to minimize possible learning effects of the inexperienced assessor during the study so that later images would not be biased by increased experience with the equipment and procedure.

To ensure adequate imaging also for the inexperienced investigators, they were introduced into ultrasound investigations by separated training sessions performed over 3 days, in which they were informed regarding crucial information to adequately perform muscle ultrasound investigations and what they have to focus to extract data such as the muscle size (orientation of fascial borders) and the PA. Image digitization and evaluation was performed by one independent assessor, blinded for the investigator. Moreover, in the training sessions, all inexperienced investigators performed between 20 and 25 images per muscle that were evaluated in the study, resulting in a minimal experience of 80 and a maximum experience of 100 acquired images before partaking in this study. To minimize learning effects, the inexperienced assessor was randomly selected from a pool of five assessors using Excel randomizer function for any given participant within this study.

#### Participant positioning and preparation

Ultrasound image acquisition was performed in the rectus femoris, vastus lateralis, gastrocnemii medialis and lateralis on two consecutive days by the experienced as well as one inexperienced assessor. Testing was performed using the right leg of the participant. Although other studies allowed several days between data collection [5, 7, 40], structural adaptations during this period (even if small) cannot be ruled out. Therefore, we tested muscles on consecutive days. After the participants were introduced to the study protocol they were placed in a seated position on a physiotherapy table. Standardization of the leg position was ensured by placing the popliteal space of the knee flush against the edge of the table with the lower legs hanging freely. Additionally, the lower-body muscles were relaxed, and a goniometer was used to ensure the knee joint and hip joint angle to be in a 90° angle. Standardization of the positioning of participants and the point to conduct the measurement was performed by both investigators together. The arms were used to stabilize the upper body to avoid any co-contractions in the hips which could affect the images. By sonographic screening of the full length of the quadriceps, the proximal (hip) and distal (knee) muscle tendon junction (MTJ) were determined by the experienced assessor which were marked with a permanent marker.

The first measurement spot for the rectus femoris was the center between the two MTJs and the second between 10 and 15 cm proximal from the distal MTJ at the knee. The variability of this second spot was used to account for differences in anatomical properties of the participants. From this second position, a horizontal line was drawn to the vastus lateralis to mark the spot at the same height on the vastus lateralis. The measurement spots on the gastrocnemius were determined similarly: The distal MTJ at the Achilles tendon was determined using an exploratory approach. At a distance of 5–10 cm (depending on individual anatomical properties of the participants) in the proximal direction the gastrocnemius medialis was marked. The gastrocnemius lateralis measurement spot was marked accordingly, albeit a bit more proximal compared to the gastrocnemius medialis due to the anatomical specificity of the gastrocnemius. This procedure was used as no intersubject comparison was conducted, so the only relevant aspect was to use the same measurement region in both testing occasions and that both assessors performed the testing at the exact same spot. That also means that the spots were re-painted at every possible instance. If any spot would have not been identifiable at any given time on these two days, the participants would have been excluded from the study. This, however, did not occur.

#### Ultrasound imaging and data processing

**Fig. 2:**
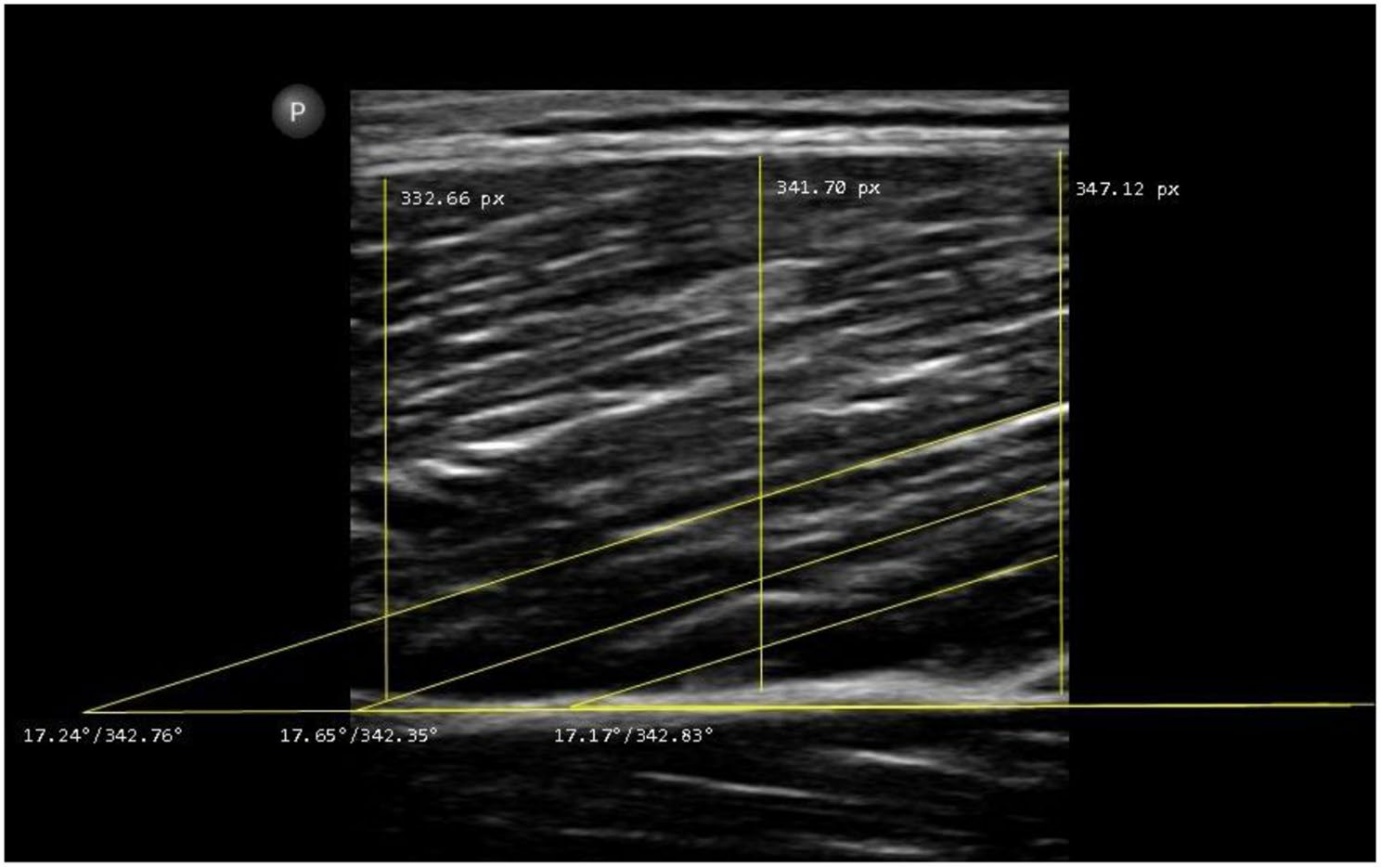
Exemplary illustration of image evaluation for muscle thickness and pennation angle for the vastus lateralis which were calculated by the ultrasound investigation software MicroDicom by drawing the angle between the fascia layer and the fascicle orientation

Randomization was performed for the assessor- and muscle-order at the first occasion using Excel randomizer function by an independent, blinded investigator. Only one assessor was present inside the lab at any given time, meaning the other entered the lab earliest once the other assessor had already finalized his image acquisition and left the room. This procedure was performed on both testing days. Ultrasound testing was performed using B-Mode ultrasound (Lumify, Software version 5.0, Philips Ultrasound LLC, Washington, USA) with a 5-cm linear probe and a frequency of up to 30 Hz. All spots were measured twice per assessor (to determine intra-assessor, intra-day(/session) reliability) on each occasion so that the mean of both could be processed for inter-day reliability evaluation, resulting in 16 images per assessor/testing occasion per participant, 32 images per participant overall and 1,248 images in total across all participants. PA and muscle thickness were evaluated by one experienced investigator blinded for test subject and assessor using MicroDicom software (Sofia, Bulgaria); [46] (Fig. 2).

### Statistical processing

Statistical analysis was performed using JASP (Version 0.18.3 (Intel), Netherlands). Normal distribution of data was checked using the Shapiro Wilk test. Mean (M) and standard deviation (SD) were stated for each muscle thickness. Reliability analysis was performed within and between days for each assessor separately. Additionally, inter-assessor reliability, also known as objectivity was evaluated between the assessors to check whether both assessors measured the same value (to be found in the Supplemental Material Table A). These analyses were conducted for relative and absolute reliability coefficients using the ICC for agreement [23] with models available

$$\:ICC={MS}_{R}-{MS}_{E}/\left({MS}_{R}+\left({MS}_{C}-{MS}_{E}\right)/n\right)$$

Where:

ICC = intraclass correlation coefficient,

$\:{\text{M}\text{S}}_{\text{C}}$ = mean square for columns,

$\:{\text{M}\text{S}}_{\text{E}}$ = mean square for error,

$\:{\text{M}\text{S}}_{\text{R}}$ = mean square for rows,

n = number of subjects,

with calculating the SEM [43],

$$\:SEM=SD*\sqrt{1-ICC}$$

where:

SEM = standard error of measurement,

SD = standard deviation of the mean difference between trial 1 and 2.

ICC = intraclass correlation coefficient.

And the MDC

$$\:MDC=SEM*1.96*\sqrt{2}$$

where:

MDC = minimal detectable change,

SEM = standard error of measurement.

These coefficients were supplemented by an agreement analysis in adherence to Bland & Altman [6, 14]. For this, the systematic bias was evaluated for significance using the paired sampled t-test [2, 19], while the qualitative error assessment was extended by quantifying absolute measurement errors via the mean absolute error (MAE) [50, 51]

$$\:MAE=\frac{1}{n}*\sum\:_{i=1}^{n}\left|{x}_{i}-{y}_{i}\right|$$

where:

*n* = number of data points.

*i* = index for each (paired) data point.

*x*_*i*_ = *i*-th data point in variable *x*.

*y*_*i*_ = *i*-th data point in variable *y*.

and mean absolute percentage error (MAPE) [21]

$$\:\text{M}\text{A}\text{P}\text{E}=\frac{1}{n}*\sum\:_{i=1}^{n}\left|\frac{{x}_{i}-{y}_{i}}{{x}_{i}}\right|*100$$

where:

*n* = number of data points.

*i* = index for each (paired) data point.

*x*_*i*_ = *i*-th data point in variable *x*.

*y*_*i*_ = *i*-th data point in variable *y*.

From the BA analysis the LoAs were extracted for each comparison while the MAE and the systematic bias were included to the graphical illustration using BA plots. The systematic bias was quantified as the mean difference and inference analysis was performed using the sampled t-test [2, 48]. The assumption to be checked was that if the evaluation was performed reliably, two measurements in a row (intra-day(/session)) or two measurements only separated by one day (inter-day/(session)) must result in one and the same value. Also, the measurements of two assessors on the same subject at the same time-point should result in the same value. ICCs were interpreted in adherence to Koo & Li [23], using the lower boundaries of the 95% CI, with ICC ≥ 0.9 being excellent. The α-level was set to 0.05.

## Results

Assumption of normal distribution was not violated in any of the parameters evaluated (*p* > 0.05). Descriptive statistics as well as reliability and measurement error quantifications of all muscles are reported in Table 1 for muscle thickness and Table 2 for PA. Both tables comprise separate sections for intra-day reliability on day 1, intra-day reliability on day 2 and inter-day reliability, depending on the assessor and muscle group.

**Table 1:** Showing descriptives and reliability statistics using the ICC, SEM, MDC, MAE, MAPE, LoAs as well as the systematic bias for the muscle thickness

	Parameter	M±SD (1)	M±SD (2)	ICC; 95% CI	SEM	MDC	MAE	MAPE (%)	LoA	Syst. Bias
	**Day 1 (Intraday)**									
**Invest. 1**	RF1/RF2	2.54±0.57	2.56±0.56	0.99; 0.98–0.99	0.003	0.007	0.04	1.48	-0.10–0.07	-0.018 (0.013)*
	VL1/VL2	2.60±0.55	2.57±0.54	0.99; 0.98–0.99	0.004	0.012	0.06	2.43	-0.13–0.19	0.029 (0.029)*
	GM1/GM2	2.02±0.33	2.02±0.34	0.98; 0.97–0.99	0.004	0.010	0.09	1.96	-0.11–0.1	-0.002 (0.770)
	GL1/GL2	1.63±0.32	1.63±0.35	0.98; 0.97–0.99	0.005	0.010	0.05	3.11	-0.12–0.13	0.006 (0.570)
**Invest. 2**	RF1/RF2	2.44±0.58	2.42±0.55	0.93; 0.86–0.97	0.020	0.060	0.12	5.39	-0.30–0.34	0.018 (0.490)
	VL1/VL2	2.55±0.51	2.57±0.56	0.96; 0.92–0.98	0.020	0.050	0.12	4.83	-0.34–0.29	-0.023 (0.380)
	GM1/GM2	2.00±0.41	2.00±0.39	0.93; 0.87–0.96	0.020	0.050	0.10	5.56	-0.29–0.29	-0.001 (0.950)
	GL1/GL2	1.59±0.37	1.62±0.39	0.93; 0.88–0.97	0.020	0.060	0.12	7.65	-0.30–0.25	-0.026 (0.260)
	**Day 2 (Intraday)**									
**Invest. 1**	RF1/RF2	2.56±0.55	2.58±0.56	0.99; 0.97–0.99	0.004	0.011	0.06	2.26	-0.18–0.15	-0.013 (0.350)
	VL1/VL2	2.58±0.53	2.56±0.54	0.98; 0.96–0.99	0.007	0.020	0.07	2.87	-0.19–0.24	0.021 (0.240)
	GM1/GM2	2.06±0.32	2.06±0.34	0.96; 0.92–0.98	0.006	0.020	0.06	3.12	-0.18–0.19	0.006 (0.690)
	GL1/GL2	1.65±0.31	1.64±0.30	0.97; 0.94–0.99	0.006	0.020	0.06	3.58	-0.13–0.15	0.008 (0.490)
**Invest. 2**	RF1/RF2	2.39±0.58	2.38±0.58	0.96; 0.93–0.98	0.030	0.050	0.12	4.84	-0.29–0.32	0.016 (0.530)
	VL1/VL2	2.49±0.53	2.51±0.50	0.95; 0.90–0.97	0.020	0.060	0.12	5.00	-0.33–0.31	-0.013 (0.620)
	GM1/GM2	2.01±0.40	1.98±0.35	0.95; 0.91–0.97	0.020	0.040	0.10	5.01	-0.20–0.27	0.035 (0.080)
	GL1/GL2	1.62±0.39	1.66±0.41	0.93; 0.87–0.96	0.020	0.060	0.11	7.04	-0.33–0.25	-0.043 (0.080)
	**Interday**									
**RF**	MEAN 1–2 I1	2.55±0.56	2.57±0.55	0.99; 0.98–0.99	0.004	0.010	0.06	2.92	-0.18–0.14	-0.018 (0.180)
	MEAN 1–2 I2	2.43±0.56	2.38±0.57	0.94; 0.90–0.97	0.024	0.070	0.14	6.34	-0.32–0.42	0.047 (0.130)
**VL**	MEAN 1–2 I1	2.58±0.54	2.57±0.53	0.98; 0.95–0.99	0.009	0.020	0.09	3.55	-0.22–0.25	0.014 (0.480)
	MEAN 1–2 I2	2.56±0.53	2.50±0.51	0.89; 0.81–0.95	0.046	0.130	0.20	8.15	-0.40–0.53	0.063 (0.110)
**GM**	MEAN 1–2 I1	2.02±0.33	2.06±0.32	0.87; 0.76–0.93	0.031	0.090	0.12	5.82	-0.37–0.29	-0.040 (0.140)
	MEAN 1–2 I2	2.00±0.39	1.99±0.37	0.88; 0.80–0.94	0.034	0.090	0.15	7.68	-0.34–0.36	0.007 (0.800)
**GL**	MEAN 1–2 I1	1.63±0.33	1.65±0.30	0.96; 0.93–0.98	0.010	0.030	0.07	4.13	-0.18–0.15	-0.017 (0.230)
	MEAN 1–2 I2	1.60±0.38	1.64±0.39	0.75; 0.58–0.87	0.068	0.190	0.19	11.70	-0.56–0.49	-0.040 (0.410)

***Legend***. I1 = experienced investigator, I2 = inexperienced investigator, Invest. 1 = experienced investigator, Invest. 2 = inexperienced investigator, GL = gastrocnemius lateralis, GM = gastrocnemius medialis, RF = rectus femoris, VL = vastus lateralis, MEAN = mean between both measures per day, M = mean, SD = standard deviation, ICC = intraclass correlation coefficient, SEM = standard error of measurement, MDC = minimal detectable change, MAE = mean absolute error, MAPE = mean absolute percentage error, LoA = limits of agreement, Syst Bias = systematic bias, * = *p* < 0.05

**Table 2:** Showing descriptives and reliability quantification using the ICC, SEM, MDC, MAE, MAPE, LoAs as well as the systematic bias for the pennation angle

	Parameter	M±SD (1)	M±SD (2)	ICC; 95% CI	SEM	MDC	MAE	MAPE (%)	LoA	Syst. Bias
	**Day 1 (Intraday)**									
**Invest. 1**	RF1/RF2	9.53±2.92	9.74±2.51	0.93; 0.87–0.96	0.14	0.39	0.75	8.10	-2.24–1.82	-0.210 (0.224)
	VL1/VL2	14.15±3.40	13.40±2.55	0.61; 0.38–0.76	0.73	2.03	1.66	14.05	-4.48–5.95	0.758 (0.088)
	GM1/GM2	23.35±3.20	22.70±2.82	0.82; 0.69–0.90	0.52	1.43	1.31	6.04	-2.92–4.16	0.619 (0.044)*
	GL1/GL2	14.11±2.15	14.58±2.05	0.86; 0.75–0.92	0.23	0.65	0.89	6.33	-2.67–1.73	-0.469 (0.014)*
**Invest. 2**	RF1/RF2	10.16±2.64	10.50±3.29	0.69; 0.50–0.82	0.71	1.96	1.80	17.48	-5.07–4.11	-0.480 (0.235)
	VL1/VL2	14.15±3.40	13.40±2.55	0.75; 0.59–0.86	0.60	1.66	1.69	15.21	-3.56–4.77	0.610 (0.101)
	GM1/GM2	22.99±2.99	23.53±3.03	0.84; 0.74–0.91	0.40	1.10	2.10	8.89	-3.89–2.59	-0.650 (0.036)
	GL1/GL2	15.35±2.76	15.86±2.63	0.72; 0.54–0.84	0.58	1.61	1.64	10.28	-4.39–3.49	-0.450 (0.201)
	**Day 2 (Intraday)**									
**Invest. 1**	RF1/RF2	9.80±2.70	9.86±2.40	0.89; 0.79–0.94	0.23	0.65	1.00	10.79	-2.26–2.26	-0.180 (0.396)
	VL1/VL2	13.80±2.83	13.56±2.58	0.86; 0.75–0.92	0.29	0.79	1.08	8.68	-2.58–3.14	0.279 (0.245)
	GM1/GM2	23.45±2.96	23.25±2.86	0.85; 0.74–0.92	0.31	0.85	1.12	4.87	-2.94–3.35	0.203 (0.439)
	GL1/GL2	14.34±2.08	14.62±1.97	0.85; 0.75–0.92	0.23	0.63	0.83	5.76	-2.42–1.87	-0.274 (0.131)
**Invest. 2**	RF1/RF2	10.60±2.54	10.81±2.56	0.68; 0.48–0.81	0.67	1.85	1.67	15.66	-4.28–3.83	-0.230 (0.529)
	VL1/VL2	13.81±2.63	13.77±2.40	0.58; 0.34–0.75	0.75	2.07	1.63	11.98	-4.55–4.63	0.042 (0.914)
	GM1/GM2	22.53±3.15	22.51±3.50	0.73; 0.57–0.85	0.76	2.12	2.08	9.56	-4.85–4.74	-0.053 (0.905)
	GL1/GL2	16.70±3.55	16.32±3.10	0.65; 0.44–0.79	0.82	2.26	3.31	16.46	-5.21–5.80	0.295 (0.557)
	**Interday**									
**RF**	MEAN RF 1–2 I1	9.64±2.67	9.80±2.50	0.97; 0.94–0.98	0.07	0.20	0.60	6.62	-1.44–1.16	-0.140 (0.210)
	MEAN RF 1–2 I2	10.40±2.71	10.70±2.35	0.73; 0.56–0.85	0.54	1.49	1.46	14.85	-3.52–3.80	-0.140 (0.670)
**VL**	MEAN VL 1–2 I1	13.78±2.69	13.68±2.62	0.91; 0.85–0.95	0.16	0.44	0.75	5.57	-2.09–2.28	0.097 (0.595)
	MEAN VL 1–2 I2	13.19±2.86	13.79±2.23	0.70; 0.52–0.83	0.65	1.80	2.92	22.09	-4.49–3.22	-0.640 (0.072)
**GM**	MEAN GM 1–2 I1	23.01±2.90	23.35±2.80	0.93; 0.88–0.96	0.15	0.42	0.80	3.53	-2.36–1.75	-0.310 (0.082)
	MEAN GM 1–2 I2	23.32±2.90	22.49±3.10	0.72; 0.55–0.84	0.66	1.82	1.70	7.62	-3.84–4.99	0.580 (0.179)
**GL**	MEAN GL 1–2 I1	14.35±2.03	14.48±1.95	0.90; 0.82–0.94	0.15	0.41	0.66	4.63	-1.88–1.62	-0.130 (0.370)
	MEAN GL 1–2 I2	15.64±2.51	16.46±3.01	0.45; 0.18–0.66	1.14	3.15	3.40	12.50	-6.31–5.05	-0.629 (0.221)

***Legend***. I1 = experienced investigator, I2 = inexperienced investigator, Invest. 1 = experienced investigator, Invest. 2 = inexperienced investigator, GL = gastrocnemius lateralis, GM = gastrocnemius medialis, RF = rectus femoris, VL = vastus lateralis, MEAN = mean between both measures per day, M = mean, SD = standard deviation, ICC = intraclass correlation coefficient, SEM = standard error of measurement, MDC = minimal detectable change, MAE = mean absolute error, MAPE = mean absolute percentage error, LoA = limits of agreement, Syst Bias = systematic bias, * = *p* < 0.05

### Muscle thickness

Overall, on day one, the ICCs indicated excellent reliability independent on the assessor and muscle evaluated (ICC = 0.93–0.99). Only for the rectus femoris and the medial gastrocnemius head the 95% CI were below 0.9 when evaluated by the inexperienced assessor, which must be, in accordance with Koo & Li [23], classified as very good. The paired sample t-test indicated significant systematic bias in the experienced assessor for the rectus femoris (*p* = 0.013) and the vastus lateralis (*p* = 0.029). However, after correcting the level of significance via FWER [44], the significant systematic bias was gone. For the experienced assessor, the SEM and MDC ranged between 0.003 and 0.005 cm and 0.007–0.012 cm, respectively, and for the inexperienced assessor there were SEMs of 0.02 and the MDC in all cases was > 0.05 cm. The LoAs for muscle evaluation of the experienced assessor ranged between − 0.1 and 0.19 cm, the mean random error remained below 3.11% (MAPE = 1.48–3.11). The LoAs in the inexperienced assessor were − 0.29–0.34cm, the MAPE was 4.83–7.65% (see Table 1).

On day two, the same classification of the ICCs was applicable, however, the 95% CI were only below 0.9 (lower limit: 0.87) in the lateral head of the gastrocnemius when images were acquired by the inexperienced assessor. For the experienced assessor, the SEM and MDC ranged between 0.004 cm and 0.006 cm and 0.011–0.02 cm, respectively, while SEMs for the inexperienced assessor were between 0.02 and 0.03; the MDC was between 0.04 and 0.06 cm. The random error quantification for the experienced assessor showed LoAs between − 0.19 and 0.24 cm with mean random errors between 2.26 and 3.58%. The inexperienced assessor exhibited LoAs ranging between − 0.33 and 0.32 with MAPEs between 4.84 and 7.04%, peaking for the lateral gastrocnemius head.

Inter-day ICCs for the experienced assessor indicated very good to excellent reliability (0.87–0.99), while the lower 95% CI boundaries were lowest for the lateral gastrocnemius head with 0.76. The SEM and MDC ranged between 0.004 and 0.031 cm and 0.01–0.19 cm. The mean random error peaked for the medial gastrocnemius head with 5.82% (see Table 1).

**Fig. 3:**
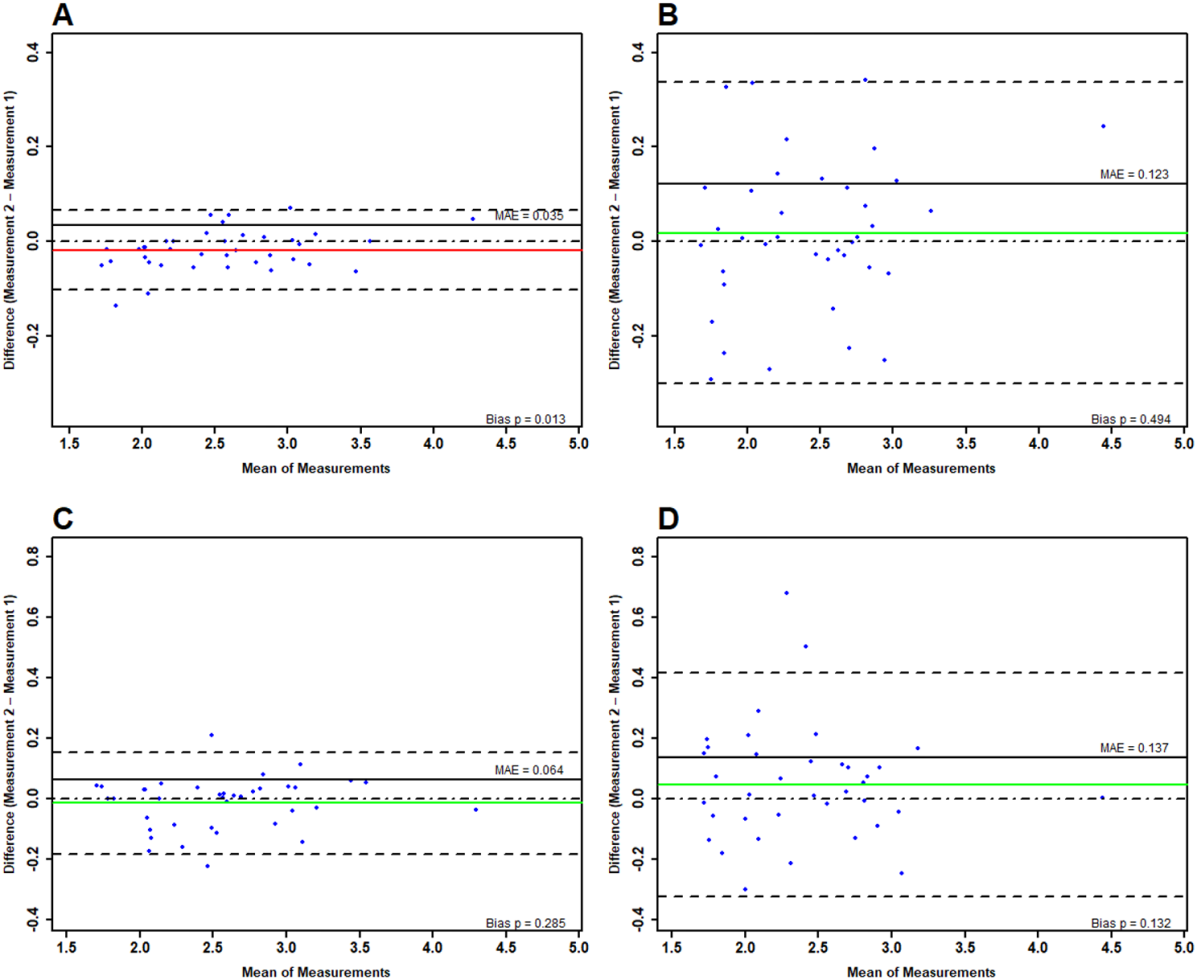
Graphical illustration of measurement errors in muscle thickness determination stemming from repeated measures between the experienced and inexperienced assessor for intra-day (**A** & **B**) and inter-day (**C** & **D**) comparisons. The green and red line represent the mean difference (green = no systematic bias, red = significant systematic bias), while the dotted lines surrounding the mean difference show the limits of agreement. The straight, black line illustrates the mean absolute error. For more extensive interpretation guidelines review [28] and [48]. The wider the limits of agreements are, the larger the random error is (indicating worse precision)

The inexperienced assessor reached ICCs between 0.75 and 0.94, with the lower 95% CI boundaries in the lateral gastrocnemius head being as low as 0.58. Absolute errors ranged between 0.024 and 0.068 cm with MDCs ranging between 0.07 and 0.19 cm. The random error reached 11.70% in the gastrocnemius lateralis, with the smallest error shown in the rectus femoris with 6.34% (see Fig. 3 for Bland Altman plots).

### Pennation angle

Relative reliability for both assessors showed moderate to excellent ICCs between 0.61 and 0.93. On day one there were SEMs and MDCs for the experienced assessor between 0.14–0.73° and 0.39–2.03° and for the inexperienced assessor between 0.40–0.71° as well as 1.10–1.96°. Random error quantification showed LoAs ranging between − 2.24–5.95° with MAPEs between 6 and 14% for the experienced assessor and LoAs between − 5.07–4.77°, with MAPEs ranging between 8.89 and 17.48% for the inexperienced assessor. After α-error correction, there were no significant systematic errors.

On day two, we found SEMs and MDC for the experienced assessor of 0.23–0.31° and 0.63–0.85°, respectively. The inexperienced assessor showed values between 0.67–0.82° and 1.85–2.26° for the SEM and MDC, respectively. The random error quantification led to LoAs between − 2.26–3.35° with MAPEs ranging between 4.87% and 10.79% for the experienced assessor, while the inexperienced assessor reached LoAs with − 4.28–5.80°. The MAPE was 9.56–16.46%.

**Fig. 4:**
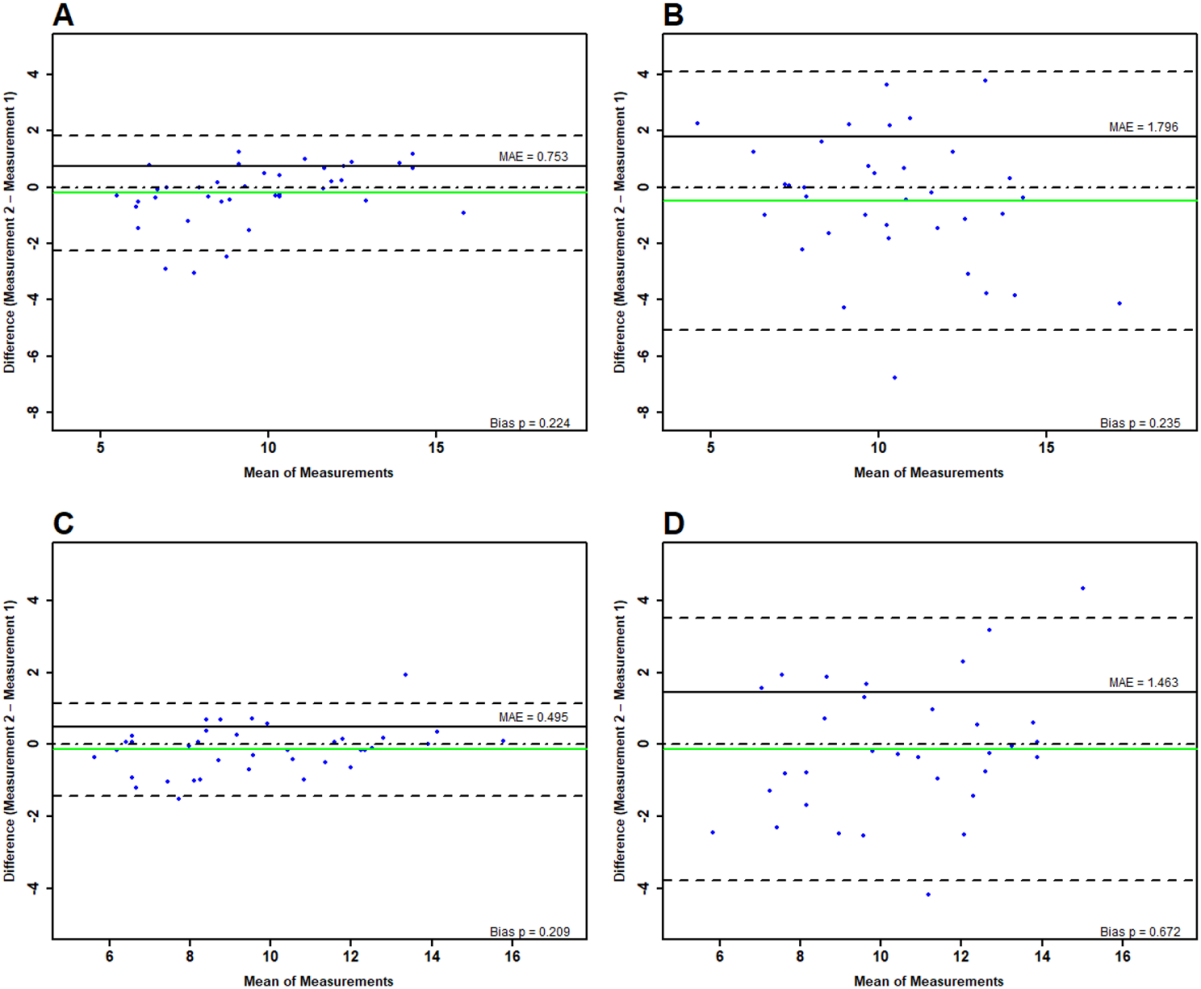
Graphical illustration of measurement errors in pennation angle evaluation stemming from repeated measures between the experienced and inexperienced assessor for intra-day (**A** & **B**) and inter-day (**C** & **D**) comparisons. The green line represent the mean difference (green = no systematic bias), while the dotted lines surrounding the mean difference show the limits of agreement. The straight, black line illustrates the mean absolute error. For more extensive interpretation guidelines review [28] and [48]. The wider the limits of agreements are, the larger the random error is (indicating worse precision)

The relative inter-day reliability was classified low to excellent, depending on the muscle evaluated and the assessor. In all cases, the experienced assessor reached very high to excellent reliability with ICCs = 0.9–0.97 (95% CI 0.82–0.98), while the inexperienced assessor showed low to moderate reliability (ICC = 0.45–0.73). Accordingly, the SEM and MDC for the experienced assessor ranged between 0.07–0.16° and 0.2–0.44°, respectively, while the inexperienced assessor evaluation indicated absolute errors with SEM = 0.54–1.14° and a MDC of 1.49–3.15°. Random errors were 3.53–6.62% for the experienced assessor and 7.62–22.09% for inexperienced assessors (see Table 2 and Fig. 4 for Bland Altman Plots).

### Inter-assessor reliability (objectivity)

Since the main objective was to investigate the influence of experience on the inter- and intra-day reliability, the inter-assessor reliability (aka objectivity) was evaluated as a secondary research question to account for clinical settings in which multiple investigators assessed the same participant/subject. Detailed results are therefore listed in the Supplemental Material Table A. In summary, although relative reliability for both, similar to the intra- and inter-day reliability, inter-assessor reliability within day one showed ICCs ranging from 0.80 to 0.87 with only a systematic error for muscle thickness in the rectus femoris (*p* = 0.01). Furthermore, muscle thickness results showed MAEs ranging from 0.15 to 0.23, corresponding to MAPEs of 7.80–11.42%. For the PA, ICCs were lower with the gastrocnemius lateralis showing the worst with ICC = 0.20, while the others ranged between 0.41 and 0.53. MAPEs were between 10.30 and 35.85%.

Similarly, on the second measurement day, the muscle thickness objectivity showed ICCs with 0.67–0.83 and for PA investigation with ICC = 0.44–0.62 with the gastrocnemius as an outlier without significant reliability (ICC = 0.03). Only for the muscle thickness evaluation of the rectus femoris and for the PA the gastrocnemius lateralis showed significant systematic bias (*p* < 0.001 and *p* = 0.004), while all other comparisons remained insignificant (*p* = 0.13–0.90). With MAPEs of 9.18–13.90% for muscle thickness and MAPE = 10.59–18.22% for the PA, the random errors were similar to those observed on day one.

## Discussion

Due to the exponential increase of studies and clinical applications that integrate muscle ultrasound imaging to explore tissue morphology, there is urgent need to explain the variance in results. A paramount relevance lies in the usage of reliable, objective and valid methods in research (and clinics) [2, 19]. Therefore, this study explored the influence of the assessor experience on intra- and inter-day reliability as a potential moderator for precision. All relative and absolute reliability indices showed better results for the experienced assessor (0.97–0.99 versus 0.93–0.96) with significant (without an overlap of 95% CIs) differences for rectus femoris, vastus lateralis and gastrocnemius medialis on day one (intra-day) and for all except the gastrocnemius medialis for the inter-day comparisons. While for intra-day on day 2 the 95% CIs between the experienced and inexperienced assessor overlapped, it must be noted that for all comparisons random errors were doubled or tripled in the inexperienced assessor. No relevant or statistically significant random errors were detected for muscle thickness investigations. Interestingly, for PA explorations, there were no significant differences for relative reliability (ICC-based statistics), but there were the same measurement caveats reflected by larger random errors, which were also reflected in doubling the range of the LoAs. These results underline the relevance of experience as a potential moderator for precision, repeatability and thus, interpretability of ultrasound investigations (with a focus on sensitive parameters such as the PA) and demonstrate the limitations of focusing on relative reliability indices [2], Barnhart et al. 2007). This study highlighted the relevance of assessor experience for ultrasound assessments, while underscoring the urgent need for detailed measurement analyses accounting for systematic and random errors (especially for inter-day reliability). Although the inexperienced investigators’ errors were much higher compared to the experienced investigator, the experienced investigator assessments cannot be seen as the gold standard because random errors also occurred indicating still limitations in standardization of the measurement and that probe pressure, angle and rotation axis might show potential for improvement. Therefore, these results should be considered to improve standardization requirements and provide scientifically sound results, which could be achieved by developing more objective and generally accepted assessment guidelines. Moreover, to improve the practical relevance and interpretability for clinicians, relevant MAE and MAPEs could potentially be used to downgrade the certainty of evidence attributed to the results of an empirical study, as they indicate that observed changes may stem from measurement variability rather than true effects of the intervention.

### Relative intra- and inter-day reliability assessment in the literature

Ultrasound muscle morphology investigation is promoted as a cost-efficient, valid and reliable method [4, 12], but those recommendations mostly stem from studies on intra-day reliability [38] or objectivity [22, 38]. Results from these studies are mostly in accordance with our results showing that relative reliability indicates satisfying reliability. For instance, a study providing ICCs that are in accordance with our data was published by Ishida et al. [20] who confirmed excellent reliability with ICC = 0.99, a SEM of 0.4 mm and a MDC of 0.1 mm (which seems surprisingly precise and we wonder if the authors have stated a wrong measurement unit) for the rectus femoris. Lanza et al. [25] explored intra-session reliability in the gastrocnemius and hip abductors in 20 middle-aged healthy participants and showed ICCs of 0.90–0.98, however with 95% CIs ranging from 0.72 to 0.99. Thoirs & English [42] reported intra-session reliability for ultrasound investigations from 18 healthy participants with 0.65–0.94. Pinto-Ramos et al. [33] determined intra-day reliability for muscle thickness in the quadriceps and indicated excellent reliability between the raters (objectivity) (ICC = 0.919–0.945) and within one rater within a day (ICC = 0.956–0.966). These individual study impressions are confirmed by few systematic reviews on the topic. Nijholt et al. [30] reviewed the literature for reliability and validity studies to quantify muscles in older adults and concluded that, overall, ultrasound was a reliable and valid measurement technique with ICC = 0.72–1.0, including 13 reliability studies, while Kwah et al. [24] described ultrasound muscle architecture investigations to be reliable, which was described as ICCs and correlation coefficients were always > 0.6.

In accordance with our results, in general, inter-day reliability indices were below those of intra-day [40], which may be partially influenced by natural biological variability (e.g. hydration level, physical activity before measurement). While Betz et al. [5] included 16 participants in their study and indicated good to excellent reliability with ICCs between 0.928 and 0.961 with 95% CIs from 0.875 to 0.978, Lima and colleagues [11] performed evaluation for rectus femoris ultrasound imaging to investigate muscle cross-sectional area and quantified relative reliability (ICC = 0.87–0.88). Santos & Armada-da-Silva [35] found high to very high ICCs for inter-session reliability (ICC = 0.81–0.99) with SEMs ranging between 0.07 and 0.19 and smallest detectable change/MDC with 0.19–0.53 cm, which is comparable with MDCs found in our study performed by the unexperienced assessors. Stausholm et al. [40] performed an extensive reliability analysis by including a reasonable sample size of 106 participants, performing inter-day, intra-day analyses and found reliability that is classified excellent (0.998 at each day), with inter-day reliability of ICC = 0.973.

Reliability, however, seems not generalizability for ultrasound investigations. Our results show dependency on the parameter (Muscle thickness or PA) as well as from the muscle evaluated. Compared to muscle thickness, there was an overall reduction in reliability for PA. On the one hand, Cronin et al. [8] showed excellent ICCs for muscle thickness (ICC = 0.99, SEM = 0.04–0.06 cm), while, on the other hand, PA showed diminished values with ICC = 0.77–0.87, SEM = 1–1.6° in 20 healthy male athletes measured in two separated occasions. Lesinski et al. [26] showed excellent relative reliability (ICC = 0.93–0.97) for muscle thickness, but PA reliability dropped to 0.41–0.49. Willemse et al. [49] explored inter-day reliability of foot muscle and plantar fascia morphology using ultrasound in 18 older adults and calculated the ICC, SEM and MDC (smallest detectable change). In line with our results, the authors showed muscle dependency with ICCs ranging from 0.57 to 0.97, lower 95% CIs starting at 0.41.

### Systematic and random error analyses

Most research focuses on relative reliability (ICC-based statistics), but some studies quantified random and systematic errors. Brusco et al. [7] compared two measurement techniques separated by 7 days in 20 participants. The authors found no systematic error for the evaluated parameters including fascicle angle and muscle thickness, while ICCs were classified very high with 0.91–0.98. Santos & Armada-da-Silva [35] found LoAs lying in the mid of those presented in this study (-0.15–0.36 versus − 0.34–0.34 and − 0.10–0.07). The poor relative reliability values for PA investigations from Lesinski et al. [26] were accompanied by no systematic error. However, the LoAs ranged from − 5.7 to 5.7, which is even worse than those of the unexperienced assessors in the presented study.

It can be therefore summarized that the reliability quantification for ultrasound investigations is as heterogenous as the way it is reported in literature. With ICC ranges starting from 0.6 [24] for muscle thickness and PA indices starting at 0.4 [26], the question arises about factors that influenced reliability. To explain variance, a special focus on the random error is worthwhile, as it suggests standardization limitations. Since the probe pressure, angle and rotation affect results of ultrasound [45], subjective influence factors such as experience are obvious moderators. Unfortunately, there are only few studies that performed a detailed measurement error analysis (only a small number of articles included LoAs) that accounted for systematic and random errors [2, 19] in addition to the ICC.

### Previous literature on the influence of experience in ultrasound evaluations

A study with a related purpose was performed by Hammond et al. [17]. The authors performed a mostly complete reliability analysis and assessed systematic bias and random error analysis through a BA analysis. However, only inter-assessor reliability was assessed, and did not evaluate the influence of ultrasound assessor experience on reliability. Another work that investigated the influence of experience was performed by Wong and colleagues [52]. The authors focused on the evaluation of forearm muscle thickness images, not on performing the muscle thickness investigation (collecting data), per se. In contrast to our study, the authors stated that less experienced assessors were able to evaluate ultrasound muscle thickness with low absolute errors. Fortin et al. [13] performed lumbar multifidus muscle imaging via ultrasound and opposed results from an experienced assessor to those of unexperienced ones. However, the authors only reported the intra-day reliability for the experienced assessor (ICC = 0.997–0.999) while focusing on inter-rater reliability/objectivity of the novice assessors. All in all, no previous study had the exact same aim as our study, making a comparison of results impossible.

By including an extensive agreement analysis, we showed that especially for sensitive parameters such as the PA, but also depending on muscles, unexperienced assessors produced comparably large random measurement errors. These errors can undermine the interpretability of changes observed over time, calling into question the reliability of conclusions in studies that fail to account for these sources of variability [48]. While systematic errors could arise from, for instance, systematically more pressure used by one assessor, the random error refers to unsystematic standardization problems within one assessor. Therefore, empirical studies must validate the reliability of their own procedures within the specific context of their research design. This includes ensuring consistent probe handling between sessions and across assessors, as well as reporting inter-session and inter-day reliability values. Without these measures, the potential for systematic or random errors could significantly limit the interpretability and reproducibility of findings. Importantly, studies using ultrasound to assess muscle properties should describe the assessors’ experience in detail, namely the approximate number of previous assessments (e.g., < 100, >500, etc.) *of each specific variable*.

Our results call for the development of standardization protocols to ensure that, especially between days (inter-day) the probe is used with the exact same pressure, angle, and rotation as performed in the previous test. To reach high objectivity, standardization procedures allowing the collecting of images with automatically applied pressure via a device [36] could have potential, so that imaging was performed without any subjectivity, comparable to MRI data collection.

### Limitations

Like every study, this work has limitations. First, it is not clear how to standardize the factor of experience. Also, assessors who performed many image acquisitions could have performed those inappropriately, while skilled assessors might provide sufficient values with less experience. Since this is a solely qualitative moderator, we are unaware of a perfect solution without limitations. Nevertheless, since especially inexperienced investigators (such as PhD students) mostly perform investigations, while experienced professors are (at least in Germany) not often involved in data collections, we think this study provides reasonable insights into measurement errors produced in such a scenario. Logically, it is not possible to provide a general guideline when an assessor can be considered experienced enough, and a detailed measurement error analysis is required for each data collection session to show reasonable reliability. Future research should evaluate critical thresholds when experience was sufficient to perform ultrasound investigations appropriately and with sufficient reliability and objectivity, which could be evaluated by correlating experience with reliability.

Another limitation is the interpretability of measurement errors. It is not clear whether we can assume the variability within the experienced investigator in the inter-day as normal biological variability or if it is a standardization problem. This normal variability can only be assessed with the gold standard, calling for a comparable study protocol using MRI assessments. In this study, we can only state that experience of the investigator causes a meaningful and clinically relevant reduction in the secondary variance, showing the relevance for precise and repeatable measurements. Nevertheless, including several experienced investigators to also assess the objectivity between these could provide further insights and could be a viable research question in future research.

## Conclusions

The study demonstrates that experience significantly (statistically and clinically) affected the intra- and inter-day reliability of muscle ultrasound evaluations. The majority of previously performed reliability studies lack quantification of random and systematic measurement errors, which are, however, of crucial importance for clinical interpretation of results, as reliability coefficients classified as excellent can be accompanied by meaningful random measurement errors. To justify measurement protocols as accurate, a detailed quantification of primary and secondary variance is necessary and authors of future studies are encouraged to account for these error sources, also if the investigator is considered experienced.

## Electronic supplementary material

Below is the link to the electronic supplementary material.

Supplementary Material 1

## Data Availability

Original data can be provided by the corresponding author due to reasonable request.
