# Supplementary material for: Examiner experience moderates reliability of human lower extremity muscle ultrasound measurement – a double blinded measurement error study
Source: Ultrasound J. 2025 Mar 26;17:20. doi: 10.1186/s13089-025-00424-6 (PMC11947354; doi:10.1186/s13089-025-00424-6)
Supplement: Supplementary file 1 — Supplementary Material 1 [file 13089_2025_424_MOESM1_ESM.docx]

**SUPPLEMENTAL MATERIAL**

Table A showing interassessor reliability for muscle thickness and pennation angle on day one and day two

|  | **Parameter** | **M±SD (1)** | **M±SD (2)** | **ICC; 95% CI** | **SEM** | **MDC** | **MAE** | **MAPE (%)** | **LoA** | **Syst. Bias** |
| --- | --- | --- | --- | --- | --- | --- | --- | --- | --- | --- |
|  | **Muscle thickness (objectivity)** | | | | | | | | | |
| **Day 1** | RF_I1 / RF_I2 | 2.55±0.56 | 2.43±0.56 | 0.86; 0.77 – 0.93 | 0.211 | 0.586 | 0.226 | 9.13 | -0.427 – 0.67 | 0.12 (p=0.011) |
|  | VL_I1 / VL_I2 | 2.58±0.54 | 2.55±0.53 | 0.87; 0.76 – 0.93 | 0.193 | 0.534 | 0.198 | 7.8 | -0.505 – 0.567 | 0.03 (p=0.492) |
|  | GM_I1 / GM_I2 | 2.02±0.33 | 1.98±0.38 | 0.84; 0.71 – 0.91 | 0.133 | 0.369 | 0.149 | 7.79 | -0.358 – 0.438 | 0.04 (p=0.231) |
|  | GL_I1 / GL_I2 | 1.63±0.33 | 1.6±0.38 | 0.81; 0.65 – 0.89 | 0.144 | 0.4 | 0.176 | 11.42 | -0.408 – 0.466 | 0.03 (p=0.427) |
| **Day 2** | R_FI1 / RF_I2 | 2.57±0.55 | 2.38±0.57 | 0.82; 0.57 – 0.92 | 0.235 | 0.651 | 0.28 | 11.6 | -0.392 – 0.76 | 0.19 (p<0.001) |
|  | VL_I1 / VL_I2 | 2.56±0.53 | 2.5±0.51 | 0.83; 0.70 – 0.91 | 0.217 | 0.601 | 0.237 | 9.18 | -0.524 – 0.656 | 0.06 (p<0.185) |
|  | GM_I1 / GM_I2 | 2.06±0.32 | 1.99±0.37 | 0.71; 0.51 – 0.84 | 0.173 | 0.48 | 0.189 | 9.61 | -0.448 – 0.577 | 0.07 (p<0.136) |
|  | GL_I1 / GL_I2 | 1.65±0.3 | 1.64±0.39 | 0.67; 0.44 – 0.81 | 0.173 | 0.479 | 0.225 | 13.9 | -0.555 – 0.574 | 0.01 (p<0.834) |
|  | **Pennation angle (objectivity)** | | | | | | | | | |
| **Day 1** | RF_I1 / RF_I2 | 9.95±2.55 | 10.4±2.71 | 0.53; 0.24 – 0.73 | 1.745 | 4.838 | 1.937 | 20.34 | -5.478 – 4.577 | 0.45 (p=0.306) |
|  | VL_I1 / VL_I2 | 13.93±2.66 | 10.4±2.71 | 0.44; 0.15 – 0.50 | 3.19 | 8.843 | 4.322 | 35.85 | -4.628 – 11.694 | 0.47 (p<0.001) |
|  | GM_I1 / GM_I2 | 23.07±2.97 | 23.19±2.97 | 0.41;0.05 – 0.66 | 2.284 | 6.33 | 2.305 | 10.31 | -6.557 – 6.312 | 0.12 (p=0.837) |
|  | GL_I1 / GL_I2 | 14.41±2.09 | 15.64±2.51 | 0.20; -0.06 – 0.55 | 1.876 | 5.199 | 2.354 | 15.57 | -6.723 – 4.256 | 1.23 (p=0.014) |
| **Day 2** | RF_I1 / RF_I2 | 9.99±2.51 | 10.7±2.35 | 0.44; 0.14 – 0.68 | 1.881 | 5.214 | 1.847 | 18.22 | -5.706 – 4.28 | 0.71 (p=0.112) |
|  | VL_I1 / VL_I2 | 13.74±2.62 | 13.79±2.23 | 0.62; 0.36 – 0.78 | 1.611 | 4.464 | 1.683 | 11.95 | -4.265 – 4.181 | 0.05 (p=0.906) |
|  | GM_I1 / GM_I2 | 23.31±2.86 | 22.49±3.1 | 0.48; 0.16 – 0.71 | 2.072 | 5.744 | 2.305 | 10.59 | -5.103 – 6.754 | 0.82 (p=0.139) |
|  | GL_I1 / GL_I2 | 14.71±1.84 | 16.46±3.01 | 0.03; -0.20 – 0.46 | 1.81 | 5.016 | 2.711 | 16.94 | -8.134 – 4.63 | 1.75 (p=0.004) |

**Legend**. CI = confidence interval, I1 = experienced investigator, I2 = inexperienced investigator, ICC = intraclass correlation coefficient,
GL = gastrocnemius lateralis, GM = gastrocnemius medialis, LoA = limits of agreement, M±SD = mean ± standard deviation,
MAE = mean absolute error, MAPE = mean absolute percentage error, MDC = minimal detectable change, RF = rectus femoris,
SEM = standard error of the mean, Syst. Bias = systematic bias, VL = vastus lateralis
